# Model potential for the description of metal/organic interface states

**DOI:** 10.1038/srep46561

**Published:** 2017-04-20

**Authors:** Nico Armbrust, Frederik Schiller, Jens Güdde, Ulrich Höfer

**Affiliations:** 1 Fachbereich Physik und Zentrum für Materialwissenschaften, Philipps-Universität, 35032 Marburg, Germany; 2 Centro de Física de Materiales(CSIC-UPV-EHU) and Materials Physics Center(MPC), 20018 San Sebastián, Spain

## Abstract

We present an analytical one-dimensional model potential for the description of electronic interface states that form at the interface between a metal surface and flat-lying adlayers of *π*-conjugated organic molecules. The model utilizes graphene as a universal representation of these organic adlayers. It predicts the energy position of the interface state as well as the overlap of its wave function with the bulk metal without free fitting parameters. We show that the energy of the interface state depends systematically on the bond distance between the carbon backbone of the adayers and the metal. The general applicability and robustness of the model is demonstrated by a comparison of the calculated energies with numerous experimental results for a number of flat-lying organic molecules on different closed-packed metal surfaces that cover a large range of bond distances.

The charge transfer at the interface between a metal and a layer of organic molecules plays a decisive role in the functionality of organic semiconductor devices and for future applications of molecular electronics. It depends crucially on the energy alignment and the wave function overlap of electronic states at such interface, which also governs the binding and even the growth of the molecular layer^1^,^2^,^3^. These interface states can either originate from localized molecular orbitals of the organic layer or from delocalized electronic states of the metal. The latter is characteristic for *π*-conjugated organic molecular layers as became first apparent for perylene-tetracarboxylic-acid-dianhydride(PTCDA) on Ag(111) for which an unoccupied, strongly dispersive interface state has been found^4^,^5^. A similar interface states has been also observed for the naphthalene-based variant NTCDA on Ag(111)^6^, for PTCDA on Ag(100)^7^ and meanwhile also for a number of other systems^8^,^9^,^10^,^11^,^12^,^13^,^14^,^15^,^16^,^17^,^18^,^19^,^20^. Time- and angle-resolved two-photon photoemission(2PPE) experiments on PTCDA/Ag(111) concluded from the dispersion and the rather short inelastic lifetime of this state that it must originate from the Shockley surface state of the bare Ag(111) substrate which is upshifted from below the metallic Fermi level by as much as 0.7 eV due to the interaction with the molecular layer^5^,^21^. This interpretation was subsequently confirmed by density-functional theory(DFT) calculations^6^,^22^,^23^,^24^,^25^,^26^, which showed that the hybridization of molecular and metallic states is rather small in the region of the projected band gap of the metal.

It turned out, however, that a realistic description of organic molecules on metal surfaces by DFT is challenging although it is one of the most widely used approaches for the determination of the geometric and electronic structure at surfaces and interfaces^27^. The large size of the organic molecules does not only require a large supercell within a slab model in the lateral direction. A reasonable description of the intrinsic Shockley surface state of the metal makes it necessary to consider also a large number of metal layers^24^. Both make such calculations very time-consuming. Moreover, metal-organic interfaces require tailored calculations methods^26^, because conventional DFT neither correctly accounts for van der Waals forces which have an important contribution to the interaction between organic molecules and metal surfaces nor for the correct long-range interaction in front of metal surfaces.

In order to highlight the main physical mechanism for the formation of the delocalized interface state at organic/metal interfaces without the help of complex DFT calculations, we propose in this letter a one-dimensional description by an analytical model potential. The choice of the potential is inspired by previous work on surface states of clean metals^28^. We recently used it for the description of image-potential states in graphene/metal systems^29^. Unlike other, more adsorbate-specific model potentials that have been used to describe interfacial electronic states,^30^,^31^ its main parameter is simply the distance of the carbon plane from the metal substrate. We will show that the same model potential not only predicts the energy of the interface state in various graphene/metal systems, but can be applied to large class of flat lying molecular layers with a similar *π*-*π* interaction as in graphene.

Our model calculation clearly illustrates how the interface state develops from the former Shockley-type surface state of the bare metal substrate with increasing interaction between the molecular film and the metal. By comparing our model results with available experimental data for different organic molecules, we show that our model is able to describe the systematic dependence of the interface state’s energy on the bond distance between the carbon backbone and the metal with predictive power if this distance and the work function are known. Moreover, the model reveals how the wave function overlap of the interface state with both, the bulk metal and the molecular overlayer depends on the carbon-metal distance.

## Results and Discussion

### Model potential

Our one-dimensional model potential is based on the nearly-free-electron model for the bulk. In this approximation, the electronic states within the metal are given by the solution of the Schrödinger equation under the influence of a weak periodic pseudopotential. Already within this simple model, the formation of the Shockley surface state can be described by introducing a potential barrier at the surface. For illustration of the basic properties of this surface state, we first recall the textbook example of a step barrier at a distance *z*_0_ from the position of the topmost atom at *z* = 0^32^,^33^. In the direction perpendicular to the surface, the potential is in this case given by


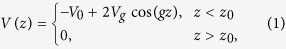


with the reciprocal lattice vector *g* = 2*π*/*a* and the distance between lattice planes *a*. With an appropriate choice of the inner potential *V*_0_ and the corrugation *V*_*g*_, this model provides a good approximation of the energy-momentum dispersion *E(k*) of electron states for the chosen direction in simple metals such as Al, Ag or Cu which are derived from *sp* electrons^34^. The model predicts an energy gap 2*V*_*g*_ in which bulk electronic states are forbidden. The bulk eigenfunctions(Bloch states) at the bottom and at the top of this gap correspond to the *k*-value at the Brillouin zone boundary 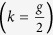, and are simply given by 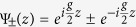. In case of a repulsive potential(*V*_*g*_ > 0), 
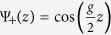
 corresponds to the upper and 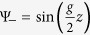 to the lower band. The corresponding eigenvalues are 
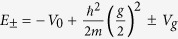
.

**Figure 1:**
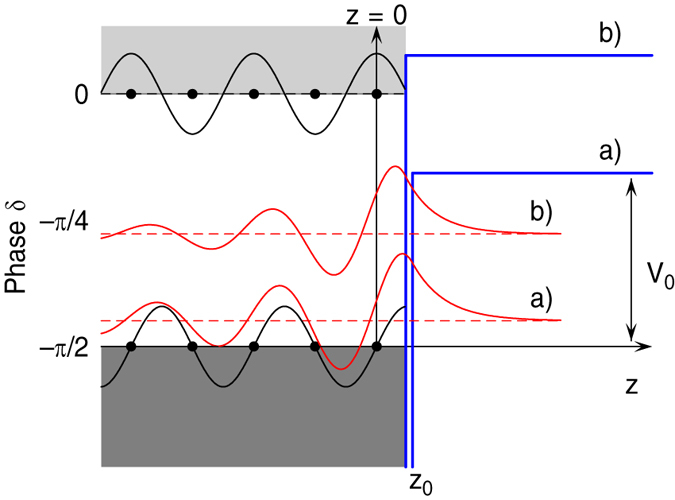
Solutions Ψ(*z*) of the Schrödinger equation for the Shockley surface state(red solid lines) for two different positions *z*_0_ and heights *V*_0_ of the surface barrier (blue solid lines). Black solid lines show the bulk solutions at the top and bottom of the bulk band gap, respectively. Black dots depict the positions of the metal atoms.

The potential barrier at the surface leads to an additional solution with an energy within the bulk band gap. The corresponding eigenfunction is located at the surface and consists of an exponentially decaying cosine Bloch function in the bulk and a simple decaying exponential on the vacuum side,


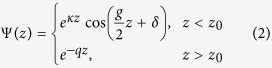


with 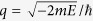 and 
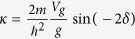
. The phase shift *δ* of the bulk cosine function is directly related to the energy *E* of the surface state. It varies from *δ* = −*π*/2 at the bottom to *δ* = 0 at the top of the bulk band gap. The wavefunctions in the bulk and in the vacuum can only be matched for one specific value of the phase *δ* or the respective energy^33^. The surface state’s energy depends on both, the height *V*_0_ and the position *z*_0_ of the surface barrier. It is, however, much more sensitive on the latter. Figure 1 illustrates how the energy of the surface state increases for increasing *V*_0_ and decreasing *z*_0_. With parameters for Ag(111)(*a* = 2.36 Å, *V*_*g*_ = 2.15 eV, *V*_0_ = 9.56 eV) and *z*_0_ = *a*/2, for example, it turns out that changing the surface state’s energy by 1 eV requires a change of the barrier height as large as 4 eV, but only a change of *z*_0_ by ∼*a*/10. The key point for the following discussion is that adlayers of organic molecules modify the distance as well as the height of the barrier, but shift the energy of the surface state predominantly according to their adsorption distance.

For our model potential we use a more realistic description of the potential barrier at metal surfaces as was introduced by Chulkov *et al*.^28^. This approach accounts for the long-range image-potential which is matched to the periodic bulk potential such that the model potential and its derivative is continuous in space. By fitting the matching parameters, not only the work function and the energies of the image-potential states, but also the energy of the Shockley surface state on a number of simple and noble metal surfaces can be quantitatively well reproduced^28^. Our model potential combines this metal potential with a potential for the molecular adlayer.

Recently, we have proposed such one-dimensional model potential for a description of the image-potential states of graphene(g) on metal substrates^29^. We could show that the energy of the image-potential states as well the coupling of their wave functions to the metal bulk systematically varies as a function of the carbon-metal distance *d*_C_. This potential is composed of four parts


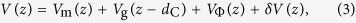


where *V*_m_(*z*) denotes the metal potential and *V*_g_(*z*) the potential of the *π*-conjugated graphene layer. The latter is a parameterized analytic expression of the potential proposed by Silkin *et al*.^35^. *d*_C_ is the distance of the carbon atoms in the graphene layer with respect to the position of the outermost metal atoms located at *z* = 0. *V*_Φ_(*z*) and *δV(z*) are corrections that consider the difference in work function between the bare and the graphene covered metal and the influence of higher-order image-charges, respectively. Beside the distance and change of the workfunction, *V(z*) is fixed by the separate properties of the metal and the adlayer and does not contain further free fitting parameters. In particular, the metal potential quantitatively reproduce not only the image-potential states, but also the Shockley surface state on the(111) noble metal surfaces.

**Figure 2:**
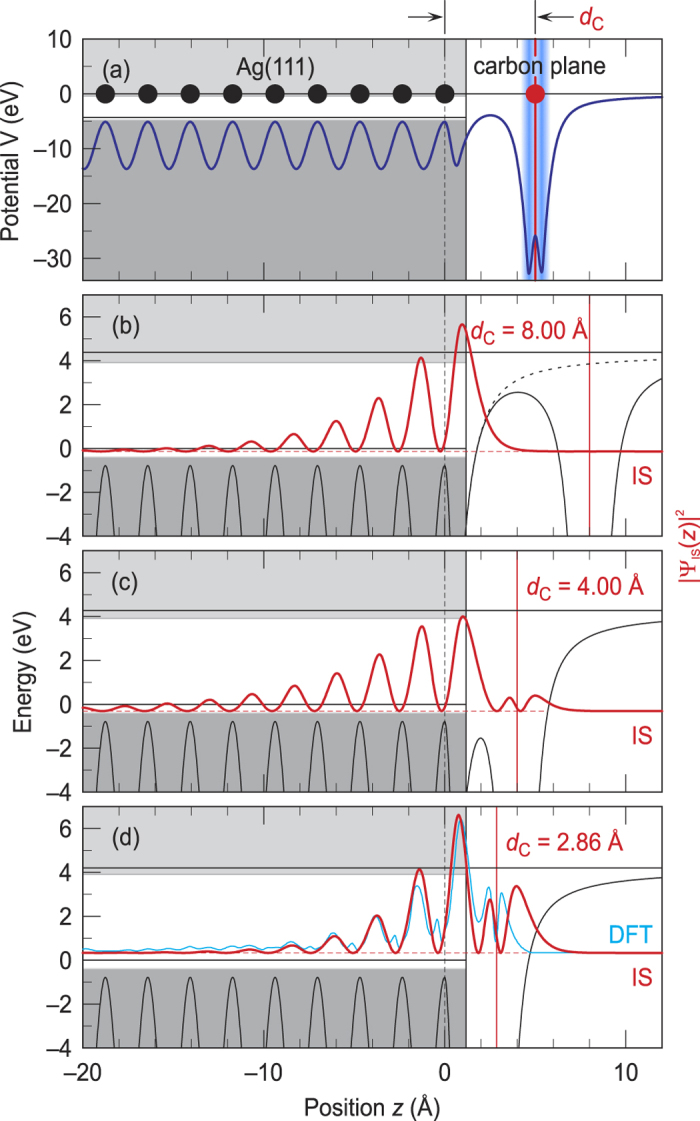
(**a**) One-dimensional model potential *V(z*)(blue solid line) for a carbon layer on Ag(111) at an exemplary metal-C distance of *d*_C_ = 5.0 Å. The positions of the uppermost Ag atomic layer and the carbon layer are depicted by vertical black dashed and red solid lines, respectively. Black and red circles illustrate the Ag and C atoms, respectively. The Ag(111) projected bulk band structure(gray shaded areas) has been extended up to the metal surface at *z*_0_ = *a*/2. The blue gradient illustrates the extension of the conjugated *π*-system of graphene^35^.(**b**–**d**) show the probability densities |Ψ_IS_(*z*)|^2^(solid red curve) of the interface state at the graphene/Ag(111) interface for metal-C distances *d*_C_ of 8.00 Å(**b**), 4.00 Å(**c**) and 2.86 Å(**d**).(**b**) additionally shows the image-potential of the bare metal surface(dotted black line). A comparison with the results of the DFT calculations for PTCDA/Ag(111)(cyan, data extracted from Fig. 4 of ref. 23) is given in(**d**).

We apply this model potential at first to a graphene layer on Ag(111) and then show that the results for this system can be directly related to adlayers of flat-lying organic molecules containing carbon rings that have a similar *π*-*π* interaction as in graphene as long as the corresponding carbon-metal distance and work function is considered. For the Ag(111) substrate, we use the parameters given in ref. 28. For the work function of the combined system, we use Φ = 4.24 eV at *d*_C_ = 3.33 Å as reported in ref. 36. *V*_g_(*z*), *V*_Φ_(*z*) and *δV(z*) are determined as described in ref. 29. Figure 2a) shows the combined potential for an exemplary carbon-metal distance of *d*_C_ = 5 Å. The wave functions Ψ and energies *E* of the Shockley surface state(SS) for bare Ag(111) and the interface state(IS) for graphene covered Ag(111) have been calculated at the center of the surface Brillouin zone(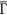-point) by solving the one-dimensional Schrödinger equation numerically by using Numerov’s method.

We characterize the coupling strength of the interface state to the metal bulk by the the fraction *p* of its probability density for *z* < *z*_0_ = *a*/2. For bare Ag(111) at the 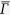-point, we calculate *p* = 76.23% and *E*_SS_ = −59 meV relative to the Fermi level.

## Discussion

In the following we discuss the transition of the Shockley state of the bare metal into the interface state upon approaching the carbon layer to the Ag(111) surface. For this purpose, we have calculated the wavefunction and energy of the former surface state for different carbon-metal distance *d*_C_. In real systems, *d*_C_ varies in the range of 2.2–3.7 Å(compare Table 1) reflecting strong and weak interaction, respectively. We start with Fig. 2b at a much larger distance of *d*_C_ = 8 Å, where the surface potential of the bare Ag(111) substrate(dotted line) is substantially modified only at distances that are larger than the extension of the Shockley surface state into the vacuum. At this distance, the probability density of the interface state(red solid line) is still basically identical with that of the Shockley surface state of bare Ag(111), but its energy relative to the Fermi level of *E*_IS_ = −136 meV is slightly reduced. This is caused by the reduction of the barrier in the region between the metal and the carbon layer as can be seen by comparing the dashed and the solid line in Fig. 2b. For smaller distances *d*_C_, there is an interplay between a further reduction of this barrier and the approach of the barrier between the carbon layer and the vacuum closer to the metal. The first leads to a decrease of the interface state’s energy, the latter to an increase. At a distance of *d*_C_ = 4 Å(Fig. 2c), which is still larger than in real systems, one can already observe the transition from the Shockley surface state of the metal to the actual interface state. Its energy of *E*_IS_ = −309 meV is, however, even further reduced. Its probability density leaks further into the vacuum and develops two small maxima around the position of the carbon layer. The overlap with the metal bulk decreases to *p* = 73.83%. For a further reduction of *d*_C_, the approach of the barrier between the carbon layer and the vacuum starts to dominate the effective barrier and results in an upshift of the interface state’s energy with decreasing *d*_C_. This case is illustrated in Fig. 2d, which shows the calculated probability density for *d*_C_ = 2.86 Å. This is just the experimental determined distance between the carbon backbone of PTCDA and the metal surface when adsorbed as a flat-lying layer on Ag(111)^37^. At this distance, the calculated energy of the interface state is now shifted substantially above the Fermi level to *E*_IS_ = +331 meV. The interface state becomes therefore an unoccupied state. The reallocation of the probability density from the metal surface into the interface and the vacuum region is even stronger as compared to *d*_C_ = 4.00 Å and the penetration into the metal further decreases to *p* = 64.70%. The blue solid line shows for comparison the laterally averaged result of a DFT calculation that has been performed for PTCDA on a nine-layer thick Ag(111) slab^24^. The agreement between our results for the one-dimensional model potential and the DFT-result is remarkably good, in particular within in metal and between the metal and the carbon plane. Above the carbon plane, however, the DFT result extends much less into the vacuum. A part of this difference might be connected to the fact that the DFT calculation does not account for the long-range image-potential that is well described by our model potential. On the other hand, our one-dimensional model does not consider lateral variations of the electronic structure which are most important within the molecular layer.

**Figure 3:**
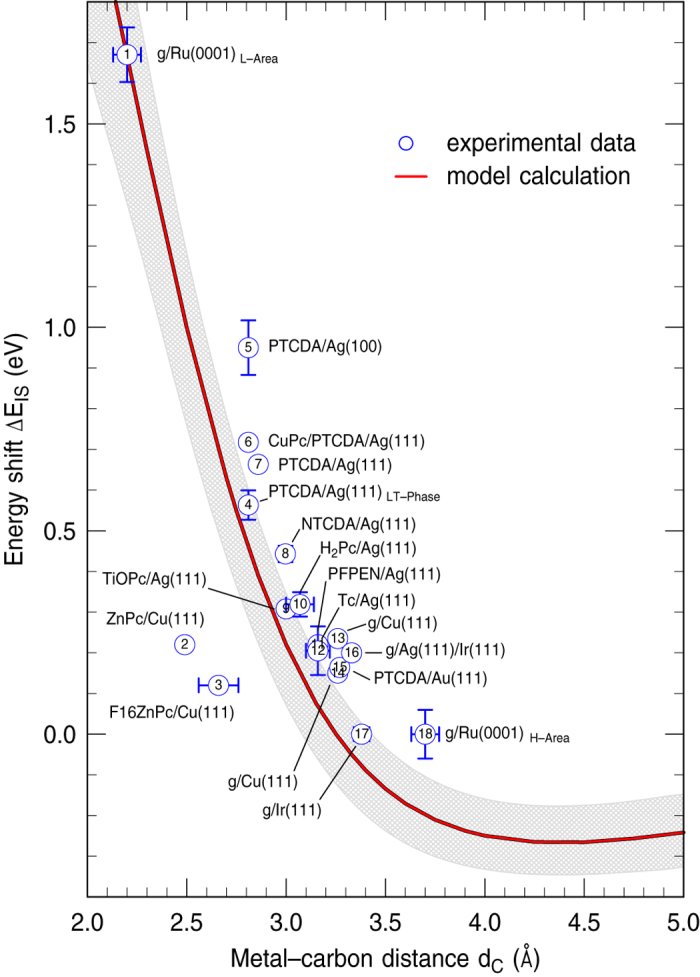
Energy shift Δ*E*_IS_ of the interface state with respect to the energy of the former surface state on the bare metal as a function of the carbon-metal distance *d*_C_. The solid red line shows the calculated results for a carbon layer on Ag(111). The gray area illustrates the variation of these results when changing the work function by ±1 eV. Symbols denote the experimental data listed in Table 1.

For a comparison with experimental results, we list in Table 1 available data on the energy difference Δ*E*_IS_ = *E*_IS_−*E*_SS_ between the interface state and the surface state as well as on the carbon-metal distance *d*_C_ for a variety of adlayers of organic molecules on metal surfaces. In order to emphasize the prototypic character of a graphene layer on a metal surface for these systems, we added also data of such systems. If available, *d*_C_ has been taken from x-ray standing wave experiments, otherwise from DFT, low energy electron diffraction or surface x-ray diffraction. Fig. 3 depicts the correlation between Δ*E*_IS_ and *d*_C_ for the experimental and the calculated results. The red solid line shows the calculated results for a work function of the combined graphene/Ag(111) system of Φ = 4.24 eV at *d*_C_ = 3.33 Å^36^. Since the work function difference between the covered and bare metal surfaces varies substantially for the different systems even at comparable *d*_C_, we depict with the gray areas the variation of the calculated results when changing the work function by ±1 eV. As can be clearly seen, the work function, i.e. the height of the potential barrier, has only a minor influence on the energy of the interface state. The latter depends instead much more sensitive on *d*_C_ and shows a strong increase for distances below ∼3.25 Å. Our model calculation can well reproduce this trend for the majority of the experimental data. We can not confirm the predicted downshift of the interface state at large *d*_C_ because this regime is not covered by real systems. The agreement between model calculation and experiment described here is rather good, even for systems like PTCDA and NTCDA on Ag(111) that are chemisorbed. A comparison with the results of DFT calculations for these two cases shows that not only the energies but also the laterally averaged wavefunctions are described well by the model^24^. The good agreement obtained for these chemisorbed organic layers is surprising only at first glance. Experiments utilizing so-called orbital tomography show that the main effect of chemisorption is simply a filling or partial filling of the former LUMO orbital^38^,^39^. Chemisorption does not result in a major distortion of the *π* system which would cause the model to break down.

Systems with stronger bonds of the functional groups of the organic molecules typically show a pronounced bending of the molecules towards the surface which reduces the carbon-metal distance at the edges^40^. Because we relate *d*_C_ to the center of the carbon backbone, this might explain the larger deviation between model and experiment for PTCDA/Ag(100). For this system, also DFT calculations^7^ clearly underestimate Δ*E*_IS_(c.f. Table 1). Even if our simple one-dimensional model gives a reasonable explanation of the main physical mechanism for the interface state formation in a number of systems, it can, however, not account for more complex chemical interactions between the substrate and the molecules. This might explain the more pronounced deviations between model and experiment for the phthalocyanines ZnPc/Cu(111) and F_16_ZnPc/Cu(111) that are subject to a stronger corrugation or distortion of the molecular layer on the substrate^41^. With the exception of these two phthalocyanines molecules, the model calculations slightly underestimate the IS energy for most systems. These small, but systematic deviations are most likely caused by molecule-metal interactions that are not taken into account by the model. Adsorption modifies the charge distribution of both the metal surface and the molecular layer. One well-known consequence is the formation of a surface dipole by the so-called cushion effect^42^ which our model takes into account via the work function in *V*_Φ_(*z*). However, also the metal as well as the graphen part of the potential will deviate slightly from the functional form of the isolated systems *V*_m_(*z*) and *V*_g_(*z* − *d*_C_) used in our model. One expects *V*_g_(*z* − *d*_C_) to become asymmetric with respect to the carbon plane and, most importantly, the position of the image-plane in the Chulkov potential *V*_m_(*z*) to move closer the substrate thus leading to an additional upshift of the IS. In order to keep the model potential parameter free we refrained from correcting for this overall small effect. A further possibility for the underestimation of the model to the experimental data is a shift of the last atomic layer of the substrate after molecular adsorption compared to the bulk values. Such a shift is not implemented in the model but it is known that the energetic position of the Shockley state depends on the lattice constant of the substrate^43^.

Finally, we note that the present one-dimensional model can, in principle, be used to also predict the effective mass of interface states, i.e. the dispersion 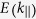 near the 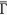 point *k*_||_ = 0. For this purpose, one simply has to solve the Schrödinger equation not only for one potential *V(z*) containing the metallic part at the 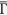 point 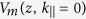, as done above, but for a whole set of 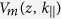. This set of potentials is readily available for all the metals included in Table 1 as their band structure is well known. One should, however, keep in mind that this approach neglects any lateral corrugation of the molecular part *V*_*g*_(*z*). It should thus be restricted to the vicinity of the 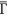 point.

## Additional Information

**How to cite this article:** Armbrust, N. *et al*. Model potential for the description of metal/organic interface states. *Sci. Rep.* **7**, 46561; doi: 10.1038/srep46561(2017).

**Publisher's note:** Springer Nature remains neutral with regard to jurisdictional claims in published maps and institutional affiliations.

## Figures and Tables

**Table 1:** Experimental data on the carbon-metal distance *d*_C_ and the energy shift Δ*E*_IS_ between the interface state and the surface state for single layers of flat-lying organic molecules and graphene layers on metal surfaces.

#	System	*d*_C_(Å)	Δ*E*_IS_(eV)
2	ZnPc/Cu(111)	2.49(3)^41^	0.22^41^
3	F16ZnPc/Cu(111)	2.66(10)^41^	0.12^41^
4	PTCDA/Ag(111)(LT-Phase)	2.81(2)^44^	0.56(3)^6^^ ‡^
5	PTCDA/Ag(100)	2.81(2)^40^	0.95(7)^7^^ †^
6	CuPc/PTCDA/Ag(111)	2.81^45^	0.72^46^^ ‡^
7	PTCDA/Ag(111)	2.86(1)^37^	0.66^5^^ ‡^
8	NTCDA/Ag(111)	3.00(2)^47^	0.44(2)^6^,^26^^ ‡^
9	TiOPc/Ag(111)	3.00(3)^48^	0.31^46^^ ‡^
10	H2Pc/Ag(111)	3.07(7)^49^	0.32(3)^50^
11	Tc/Ag(111)	3.16^43^	0.22^51^
12	PFPEN/Ag(111)	3.16(6)^52^	0.21(6)^46^^ ‡^
15	PTCDA/Au(111)	3.27(2)^53^	0.164(4)^11^
1	g/Ru(0001)(L-Area)	2.20(7)^54^,^55^,^56^,^57^	1.67(7)^54^^ †^
13	g/Cu(111)	3.26^36^	0.24^58^^ ‡^
14	g/Cu(111)	3.26^36^	0.15^59^
16	g/15ML-Ag(111)/Ir(111)	3.33^36^	0.20^60^
17	g/Ir(111)	3.38(4)^61^^, *^	0.00^62^
18	g/Ru(0001)(H-Area)	3.70(7)^54^,^55^,^56^,^57^	0.00(6)^54^^ †^
	PTCDA/Ag(100)(DFT)	2.81(2)^40^	0.63^7^^ †^
	PTCDA/Ag(111)(DFT)	2.86(1)^53^	0.56^24^
	NTCDA/Ag(111)(DFT)	3.00(2)^47^	0.32^24^

The numbering corresponds to that of the data points in Fig. 3 in addition, results of DFT calculations are listed for NTCDA and PTCDA on Ag surfaces.(Annotations: ^†^energy relative to the one of the respective surface resonance of the bare substrate, ^‡^relative to energy of Shockley state of the bare substrate from ref. 63, *average distance).
